# Presence of plaque, gingivitis and caries in Sudanese children with congenital heart defects

**DOI:** 10.1007/s00784-016-1884-2

**Published:** 2016-06-24

**Authors:** Hiba Mohamed Ali, Manal Mustafa, Siham Hasabalrasol, Osama Hafiz Elshazali, Elwalid Fadul Nasir, Raouf Wahab Ali, Ellen Berggreen, Marit Slåttelid Skeie

**Affiliations:** 1 Department of Clinical Dentistry, Faculty of Medicine and Dentistry, University of Bergen, Årstadveien 19, 5009 Bergen, Norway; 2 Oral Health Centre of Expertise in Western Norway, Bergen, Hordaland Norway; 3 Ahmed Gasim Hospital, Khartoum Teaching Hospital, Khartoum, Sudan; 4 Ahmed Gasim Hospital, Faculty of Medicine University of Khartoum, Khartoum, Sudan; 5 Department of Periodontics, University of Science and Technology, Khartoum, Sudan; 6 Department of Biomedicine, Faculty of Medicine and Dentistry, University of Bergen, Bergen, Norway

**Keywords:** Caries, Plaque, Gingivitis, Children, Congenital heart defects

## Abstract

**Objectives:**

The objective of this study is to assess the presence of plaque, gingivitis, and caries in a group of Sudanese children with congenital heart defects CHDs (cases) and compare them to children without CHDs (controls).

**Materials and methods:**

This analytical cross-sectional study included cases (*N* = 111, with a mean age of 7.2 ± 3.0 years) and controls (*N* = 182, with a mean age of 7.2 ± 2.8 years) from Khartoum, Sudan. Examinations were done by two calibrated dentists using plaque index, gingival index, and WHO (World Health Organization) caries diagnostic criteria (dmft/DMFT index: decayed, missing, and filled teeth).

**Results:**

Children with CHDs (cases) had statistically significantly higher mean number of sites with plaque and gingivitis than children without CHDs (controls), although almost all children experienced plaque. Cases also experienced significantly higher mean dmft/DMFT than controls (age group 1, 3–7 years: 3.7 vs 2.3 and age group 2, 8–12 years: 1.3 vs 0.6). The Significant Caries Indices in cases (age groups 1 and 2) were also significantly higher than among controls (SiC 8.2 vs 5.9 and 1.8 vs 0.8, respectively). Fillings were totally lacking among cases and scarce among controls.

**Conclusions:**

The findings clearly showed that this group of Sudanese children with CHDs was more severely affected with gingivitis and caries than the control group without CHDs. These results are cause for concern in children at risk of developing systemic infections and serious complications related to poor oral health.

**Clinical relevance:**

These findings provide important baseline data for planning appropriate dental preventive strategies for Sudanese children with CHDs.

## Introduction

Congenital heart defects (CHDs) are congenital defects that occur during the embryonic development of the heart or the great vessels, resulting in functional impairment of both heart and circulation [1]. CHDs account for 28 % of all major human congenital defects [2]. The global incidence is estimated to be eight per 1000 [3, 4]. A systematic review from 2011, which included 114 papers, disclosed a substantial increase in the prevalence of CHDs during the period 1930–1995, from 0.6 per 1000 to nine per 1000 [3]. In Sudan, a study carried out in 2007 reported that the prevalence of CHDs among Sudanese children aged 5–15 years was 2.0 per 1000 [5]. In 2013, ventricular septal defect (VSD), atrial septal defect (ASD), patent ductus arteriosus (PDA), pulmonary stenosis (PS), and tetralogy of Fallot (TOF) were the most common CHDs and they accounted for 83 % of all congenital heart defect cases in Sudan [6].

As a result of progress in medical procedures and the use of prosthetic materials to correct CHDs, the prognosis has improved. Children with CHDs now have a greater life expectancy, and many of them reach adulthood [7]. Nevertheless, these children remain at a higher risk than other children of developing serious and potentially fatal heart-related infections, such as infective endocarditis [7]. Infective endocarditis of oral origin has conventionally been thought to be associated only with invasive dental procedures, through the entry of oral bacteria into the systemic circulation (for example, *Streptococcus viridans*, *S. sanguinus*, and *S. mutans*) [8]. However, later studies have shown that it can be caused by the everyday bacteraemia associated with routine daily activities such as chewing, flossing, and tooth brushing [9]. Moreover, children with CHDs are also susceptible to the development of various oral diseases [8]. Several factors, independently or in combination, promote this susceptibility to oral disease, including: (1) malnutrition and growth retardation, frequently with extra meals to compensate, especially during the night [10], (2) long-term medication with antibiotics sweetened with sucrose [11], (3) impaired salivary flow induced by CHD drugs [12], and (4) lack of parental attention to their child’s oral health due to the parents’ concern for their children’s general health [13]. Studies of gingival and periodontal health in this group have also disclosed more severe gingival diseases and more accumulated plaque [14] than in a healthy group. Additionally, a recent study has shown that the enamel and dentin structures of the deciduous teeth in children with CHDs are structurally and chemically altered, with low calcium and phosphorus levels [15].

With the growing concerns about the oral problems related to CHDs, several studies in different countries have compared the caries experience of children with CHDs to that of those without CHDs. In some of these studies, the prevalence of caries and gingivitis among children with CHDs was much higher than in their counterparts [13, 16, 17]; however, other studies failed to demonstrate such differences [18, 19]. One study even reported better oral health parameters among children with CHDs than among other children [20].

Sudan is regarded as an underdeveloped country, with a large population and a low average income. The number of dentists in the population is very low, estimated at 1 per 100,000 people [21]. With the limited resources and the lack of systematized dental preventive programs, the availability of dental care for all children, and especially those with chronic diseases, is expected to be quite limited [21].

In Sudan, some epidemiological studies have been carried out focusing on different aspects of children’s oral health status [22–26], but none has yet included children with CHDs. Therefore, it is not yet known whether Sudanese children with CHDs have worse or equivalent oral health when compared with Sudanese children without CHDs. The aim of the present study was, therefore, to assess the presence of plaque, gingivitis, and caries in a group of Sudanese children with CHDs (cases) and to compare them with children without CHDs (controls). The hypothesis to be tested was that plaque, gingivitis, and caries are more prevalent in the former group.

## Materials and methods

### Study design

This was an analytical cross-sectional study among a group of children affected by CHDs (“cases”), compared with children without CHDs (“controls”). The study was conducted in two phases: from January to August 2011 and from March to September 2014. For the cases, ethical approval was obtained from the Ahmed Gasim Hospital, from the Sudanese Federal Ministry of Health, the Research Ethics Committee at the University of Science and Technology and from the Regional Committee for Medical Research Ethics, Western Norway (No. 2265). For the controls, ethical approval was obtained from the State Ministry of Education (Khartoum) and the State Ministry of Primary and Pre-school Education in the three Khartoum districts. Ethical consent forms were also obtained from the local offices of the Ministry of Education in each of the selected localities. These consent forms were taken to each of the selected schools. Confidentiality was ensured for both cases and controls. Consent forms were completed and signed by the guardians of the study participants after obtaining verbal agreement.

### Study population

The sample size calculation was conducted by using the two-sided Student sample *t* test (the test to be used for comparisons). The smallest difference to be detected in the mean (dmft/DMFT: decayed, missing, and filled teeth) between the two groups was 1 and the variance was estimated to be 2.0 in the controls and 2.5 in the cases. The level of significance was set at 0.05 and the power at 80 %. The estimated sample size was 60 cases and 60 controls in each age group: age group 1 (3–7 years) with deciduous teeth and age group 2 (8–12 years) with permanent incisors and first molars. Altogether, the estimated sample consisted of 240 participants in both case and control groups.

Cases were recruited from The Ahmed Gasim Cardiac Center in Khartoum, Sudan. The inclusion criterion was a confirmed diagnosis with a CHD in the age group 3–12 years, critically ill children, and those using medications other than for CHDs were excluded. Participants in the control group were recruited from schools and kindergartens in the Khartoum state. A stratified random sampling technique was used. The strata were rural–urban, with group matching of cases and controls in terms of age and sex. About 60 controls from each of the three districts of the Khartoum state (the districts: Khartoum City, Khartoum North, and Omdurman) were enrolled. According to the State Ministry of Primary and Pre-school Education, Khartoum City is divided into six localities (all urban), Khartoum North into five localities (three urban and two rural), and Omdurman into five localities (three urban and two rural). One rural and one urban locality were randomly selected from each of three districts (simple random sampling by draw) in Khartoum North and Omdurman and two urban localities were selected from Khartoum City. Thereafter, from each selected locality, one male school, one female school, and one kindergarten were randomly selected (simple random sampling from the list of the schools in each selected locality). Further, children were randomly selected from each of the schools (grade 1–grade 8 every tenth child). Children were excluded from the study if the consent form was not signed by one of the parents. The response rates among cases and controls were 94.8 and 95.7 %, respectively. Altogether, 117 cases and 190 controls were invited to participate, a total of 111 cases and 182 control participated, age group 1 with 62 cases and 101 controls and age group 2 with 49 cases and 81 controls.

### Calibration and reliability tests

Two dentists, examiners 1 and 2 were trained and calibrated in detection of caries and detection of gingival inflammation and plaque (both theoretical and practical training) according to indices used by a dental pediatric specialist through the examination of 10 children (4–12 years). Cohen’s kappa test was used to measure inter- and intra-examiner reliability. Test I was the inter-examiner caries reliability test between examiners 1 and 2 based on the examination of all teeth of 20 children (6–12 years) for the detection of caries. Tests IIa and IIb measured intra-examiner reliability for caries detection by examiners 1 and 2, respectively, based on the examination of ten children for examiner 1 (test IIa) and another ten children for examiner 2 (test IIb) (6–12 years). Each examiner examined all teeth and the scores were recorded in clerking sheets. The same children were re-examined within a week interval and the new scores were recorded. Tests III and IV measured the inter-examiner agreement between examiners 1 and 2 with respect to the detection of gingivitis and plaque. This was based on the examination of the six index teeth for gingival index (GI) (test III) and for plaque index (PI) (test IV) of 20 children (6–12 years). The respective inter-examiner Cohen’s kappa values for tests I, III, and IV were 0.6, 0.6, and 0.6. The intra-examiner reliability tests, IIa and IIb, showed Cohen’s kappa values of 0.9 and 0.9.

### Dental examinations

The data were based on clinical examination of the teeth and gingivae. Clinical dental examinations were conducted in a separate room close to the referral room, with good daylight. All children were seated in regular chairs. The examiners registered caries by using a plain mouth mirror and dental probe (explorer), following the WHO criteria and scoring system: caries was registered as lesion in the pits and fissures, in a smooth tooth surface with an unmistakable cavity, undermined enamel, or detectable softened floor or wall, destroyed crown, temporary fillings, and permanent fillings with secondary caries [27]. Decayed, missing, and filled teeth (dmft/DMFT) indices were used. In age group 1, the dmft data were based on all primary teeth, while for age group 2, the DMFT data were derived from permanent central and lateral incisors and the first permanent molars. The Significant Caries Index (SiC) was additionally calculated for age groups 1 and 2, using the online calculation of SiC from Malmö University, presenting the mean dmft /DMFT of the one-third of the participants with the highest dmft/DMFT scores [28].

Gingivitis was measured using a simplified form of the gingival index (GI) [29]. This index measures the site prevalence, indicating the number of affected sites in one patient. Six sites were examined (55/16, 51/11, 65/26, 75/36, 71/31 and 85/46). Dichotomous scoring was used, where visual inflammation and a tendency to spontaneous bleeding was scored as 1, while the absence of these signs was scored as 0. For comparison between the cases and the controls, both the mean number of sites affected with gingivitis and the number of individuals in the sample (as percentages) with at least one site with gingivitis were used.

The presence of plaque was measured using a simplified form of the plaque index (PI) [29]. A probe was pressed into the gingival margin parallel to the buccal tooth surface and six sites were examined (55/16, 51/11, 65/26, 75/36, 71/31, and 85/46). The presence of visible plaque on at least one surface was given a score of 1 and the absence of plaque was given the score 0. Both the mean number of sites with detectable plaque and the number of individuals out of the sample (percentages) with at least one site with plaque were used.

The entire oral cavity was also examined for the presence of any ulceration, trauma, abnormal discoloration, discharging sinuses, or swelling. Finally, gingival overgrowth was recorded using the simplified gingival overgrowth index, according to Miller and Damm, 1992 [30]. Participants with gingival overgrowth covering more than one third of at least one tooth were given a score of 1, while participants without overgrowth were given a score of 0.

### Statistical methods

After data entry and review, statistical analyses were undertaken using SPSS version 22. Descriptive statistics included mean and standard deviations (SD) for continuous variables and frequency and percentages for categorical variables. A Student’s *t* test was used to compare means between groups, and the non-parametric Mann–Whitney test was applied for skewed data. The chi-square test and Fisher’s exact test were used to compare differences in percentages between groups. Stratified analyses were not considered necessary, because the sampling fractions within the strata were very similar. Logistic regression analysis was used to control for possible differences in socio-demographic variables between the groups, through the calculation of the odds ratio (OR) with 95 % confidence interval. The level of statistical significance was set at 0.05, with a confidence interval of 95 %.

## Results

The mean age for all cases (62 girls and 49 boys) was 7.15 (SD 3.0) years, and it was 7.19 (SD 2.9) years for the controls (89 girls and 93 boys). Cases were diagnosed with a range of CHDs, among which VSD, ASD, PDA, PS, and TOF were the main types reported, accounting for about 70.0 % of all diagnoses. The other types were combinations of these and there were small percentages of cases with rare conditions associated with CHDs, including Noonan’s syndrome and Marfan’s syndrome.

No significant differences were observed between the cases and the controls with regard to the socio-demographic data, except for the mothers’ education in age group 1 (Table 1). However, the mother’s level of education did not affect the caries experience of their children (OR 1.5 (CI 0.7–3.1), *p* > 0.05). The prevalence of both gingivitis and caries was significantly higher among cases than controls, as seen in Table 2. Almost all children, both cases and controls, had at least one site with visible plaque. However, the mean number of sites with detectable plaque (4.9 (SD 1.7) among the cases vs 3.8 (SD 1.9) among the controls) and the mean number of sites with gingivitis (4.1 (SD 2.3) among the cases vs 1.9 (SD 1.9) among the controls) were both significantly higher among the cases than the controls. Moreover, among the cases, gingival overgrowth was recorded in 13.5 %, compared with 1.0 % among the controls. Abscesses and discharging sinuses associated with carious teeth were recorded in 2.0 % of the cases while there were none in the controls. Two cases with excessive salivation were recorded, both with Down’s syndrome.

**Table 1:** Frequency distribution of the socio-demographic and dental clinical variables between cases (CHD) and controls within age group 1 (cases (62) and controls (101)) and age group 2 (cases (49) and controls (81)) in numbers and percentages

Socio-demographic	Age group 1 (3–7 years, deciduous teeth)			Age group 2 (8–12 years, permanent teeth)		
Sex	Male (%)	Female (%)	*p* value	Male (%)	Female (%)	*p* value
CHD Controls	26 (41.9) 50 (49.5)	36 (58.1) 51 (50. 5)	0.419	23 (46.9) 39 (48.1)	26 (53.1) 42 (51.9)	0.470
Mother’s education	Illiterate (%)	Primary, secondary, university (%)		Illiterate (%)	Primary, secondary, university (%)	
CHD Controls	12 (19.4) 3 (3.0)	50 (80.6) 98 (97.0)	0.002*	10 (20.4) 7 (8.9)	39 (79.6) 72 (91.1)	0.056
No. of rooms	1–2 (%)	3–4 or more (%)		1–2 (%)	3–4 or more (%)	
CHD Controls	34 (54.8) 57 (56.4)	28 (45.2) 44 (43.6)	0.485	29 (59.2) 50 (63.3)	20 (40.8) 29 (36.7)	0.390
Marital status (mother)	Married (%)	Divorced or widowed (%)		Married (%)	Divorced or widowed (%)	
CHD Controls	58 (93.5) 100 (99.0)	4 (6.5) 1 (1.0)	0.075	40 (81.6) 70 (89.7)	9 (18.4) 8 (10.3)	0.150
Mother’s occupation (%)	Employed (%)	Unemployed (%)		Employed (%)	Unemployed (%)	
CHD Controls	1 (17.7) 21 (20.8)	51 (82.3) 80 (79.2)	0.396	8 (16.3) 21 (26.6)	41 (83.7) 58 (73.4)	0.129

The chi-square test was used to test for differences in prevalence (percentages) between groups

*χ ^2^ test significant *p* < 0.05

**Table 2:** Comparisons of prevalence of plaque, gingivitis, and dental caries and comparisons of the mean number of sites with plaque, gingivitis, and mean dmft/DMFT in cases (CHD) and controls within age groups 1 and 2

Total (3–12 years)	CHD (*N* = 111)	Controls (*N* = 182)	*p* value
	%		
Plaque present (PI ˃ 0)	91.8	93.7	0.651
Gingivitis (GI ˃ 0)	82.0	64.8	0.002**
Caries (dmft ˃ 0, DMFT > 0)	66.7	46.7	0.001**
Age group 1 (3–7 years)	CHD (*n* = 62)	Controls (*n* = 101)	*p* value
	%		
Plaque present (PI ˃ 0)	90.3	87.1	0.533
Gingivitis (GI ˃ 0)	72.6	51.5	0.001**
Caries (dmft ˃ 0)	77.4	56.5	0.007**
	Mean (SD)		
Plaque present	4.7 (1.9)	3. 3 (1.9)	0.001**
Gingivitis	3.5 (2.5)	1. 4 (1.7)	0.001**
dmft	3.7 (3.8)	2.3 (3. 2)	0.021*
SiC	8.2	5.9	0.008**
Age group 2 (8–12 years)	CHD (*n* = 49)	Controls (*n* = 81)	*p* value
	%		
Plaque present (PI ˃ 0)	98.0	97.5	0.875
Gingivitis (GI ˃ 0)	93.9	81.5	0.066
Caries (DMFT ˃ 0)	53.1	34.6	0.038*
	Mean (SD)		
Plaque present	5.2 (1.5)	4.5 (1.7)	0.006**
Gingivitis	4.8 (1.8)	4.8 (1.8)	0.001**
DMFT	1.3 (1.7)	0.6 (0.9)	0.008**
SiC	1.8	0.8	0.001**

The chi-square test was used to test for differences in prevalence (percentages) between groups. Dichotomous variables were used (PI > 0: children with plaque in at least one site, GI > 0: children with gingivitis in at least one site, dmft > 0/DMFT > 0: children with caries in at least one tooth). *t* test was used for the comparisons of the mean number of sites plaque and gingivitis. For the comparisons of the mean dmft for primary teeth and DMFT for permanent teeth, Mann–Whitney test was used

*Cases* children with congenital heart defects, *controls* children without congenital heart defects, *N* number of participants in each age group

Significant *p < 0.05 and **p < 0.01

### Age group 1 (primary dentition)

**Fig. 1:**
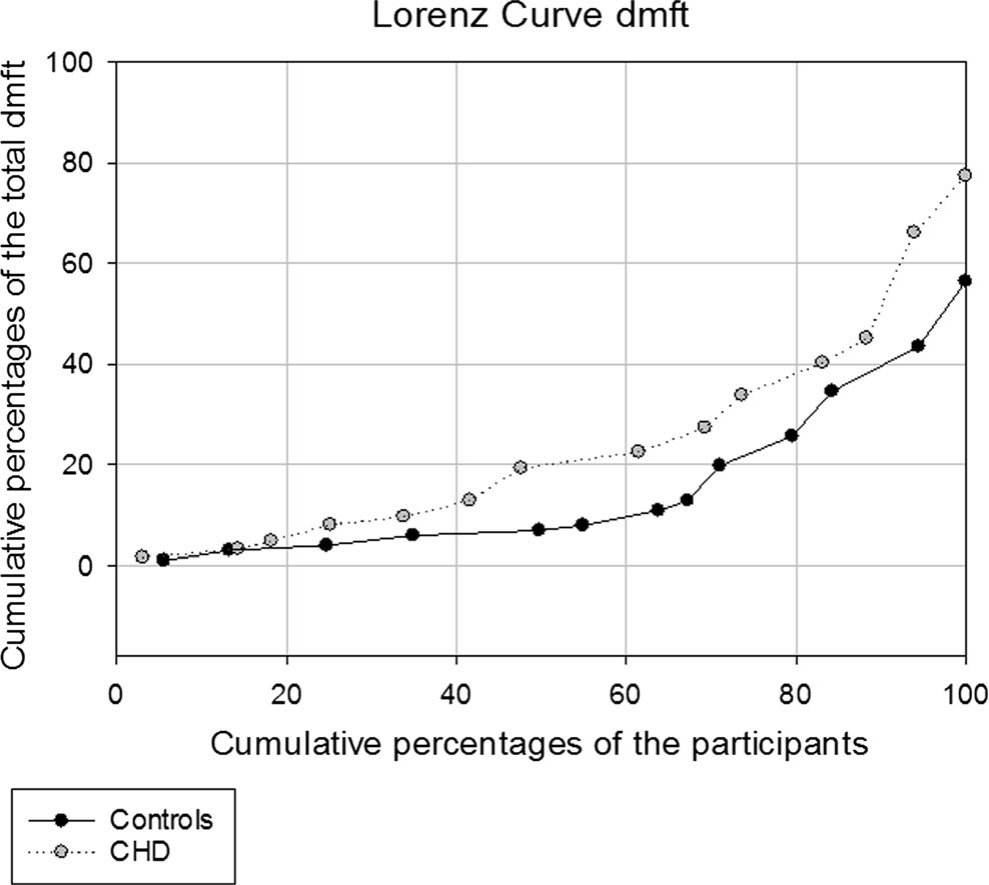
Lorenz curve for caries distribution (dmft) in age group 1 (3–7 years) in a sample of Sudanese children with and without CHD

The mean age of the participants was 4.8 (SD 1.4) years for the cases and 4.9 (SD 1.4) years for the controls. The prevalence of plaque, gingivitis, and caries in cases and controls is presented in Table 2. There was no statistically significant difference between the groups with respect to the number of individuals with at least one site with plaque, but a significantly higher percentage of cases had at least one site with gingivitis (Table 2). Caries was to some extent skewed among both cases and controls, but was more widely disseminated among the cases (75.0 % of all caries lesions in the cases were recorded in 34.8 % of the participants, whereas the same proportion of all caries lesions occurred in 26.0 % of the controls) (Fig. 1). As seen in Table 2, caries affected more than two thirds of the cases compared to almost half of the controls. The mean dmft values were significantly higher among the cases compared to controls and the Significant Caries Index (SiC) for the cases was especially high (Table 2). The mandibular anterior deciduous teeth had caries in both groups (12.9 % of the cases vs 9.9 % of the controls). The decay component (dt) constituted more than 95.0 % of the dmft values and fillings were recorded in only five controls and in none of the cases. The mean number of sites with gingivitis and detectable plaque were significantly higher among the cases than among the controls (Table 2).

### Age group 2 (index permanent teeth)

**Fig. 2:**
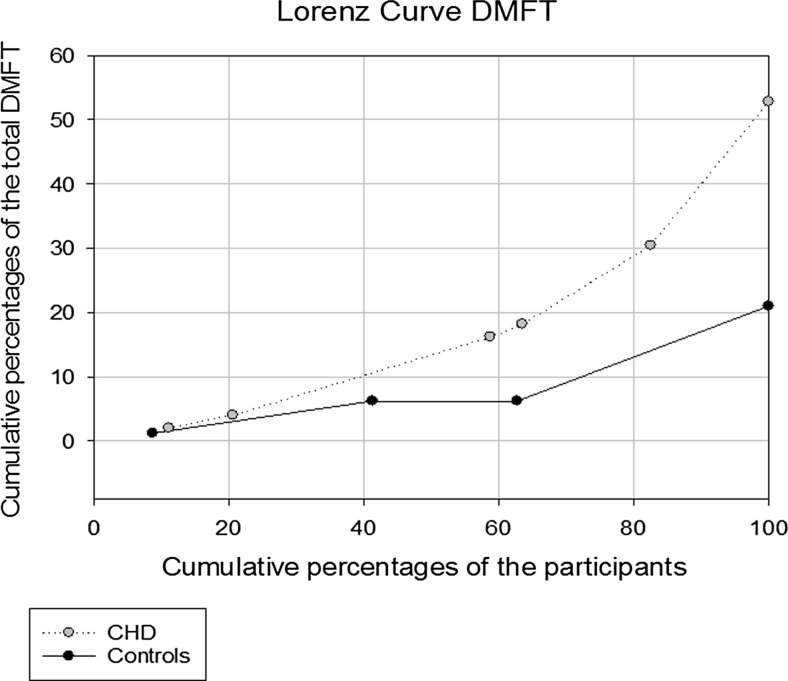
Lorenz curve for caries distribution (DMFT) in age group 2 (8–12 years) in a sample of Sudanese children with and without CHD

As shown in Table 2, when compared with the controls (mean age 10.0 (SD 1.3) years), plaque, gingivitis, and caries were more prevalent among the cases (mean age 10.1 (SD 1.3) years). Over 95.0 % of both controls and cases had at least one site with plaque, without statistically significant differences. The mean number of sites with gingivitis and the mean number of sites with detectable plaque were statistically significantly higher among the cases (Table 2). Half of the cases, compared with one third of the controls, were affected by caries on the permanent index teeth (Table 2). The mean DMFT values were significantly higher among the cases than the controls (Table 2). The decay component (DT) constituted the major portion (about 95.0 %) of the DMFT value. The filling component (FT) was again entirely absent among the cases and extremely low among the controls. Figure 2 shows the distribution of caries (DMFT) among the cases and controls.

## Discussion

Epidemiological studies focusing on the oral health of children with CHDs are necessary to provide baseline data for planning future preventive strategies. Few epidemiological studies have focused on the oral health of children in Sudan, and this was the first to be conducted among children with CHDs. The main finding was that children with CHDs constituted a disadvantaged group with respect to the oral health indicators examined. The findings illustrated that the SiC index was almost as twice as high among children with CHDs than in the controls. Almost all the carious teeth recorded in both groups represented untreated lesions in primary teeth, and even mandibular anterior teeth were affected, which is an indication of high caries activity among cases [31]. These findings are concerning because these children are at risk of developing infective endocarditis [8]. For children with CHDs in particular, poor oral health should be avoided because the complications represent a continuous threat to their lives [8]. As previously documented, caries in the primary dentition increases the likelihood of developing future caries in the permanent dentition [32]; in addition, plaque and gingivitis during childhood may give rise to periodontitis later in life [33].

Poor oral health among Sudanese children may be explained to some extent by the general attitude of the Sudanese towards dental care and the low awareness of the importance of dental preventive measures [21]. In 2009 it was demonstrated that most of the dental visits made by the Sudanese population were associated with caries and dental pain [34]. A later study in 2012 indicated marked inequalities and disparities in the provision of dental care and showed that the majority of the population had restricted access to regular dental care [21]. People only sought dental treatment when they experienced severe pain or swelling, and the most common treatment was tooth extraction [21]. In that study, low dental attendance among Sudanese people was explained by the relatively low number of dentists available, resulting in a lack of accessibility and availability of regular dental care, lack of public funding and insurance coverage for most dental treatment modalities, and the high costs of the treatment available [21]. It is therefore, a crucial need for improvements within the dental health care systems and the provided services especially for the high risk children including those with CHDs. Preventive programs should be implemented through the proper education of caregivers about the importance of regular preventive dental routines and visits. Specialized trained dental staff for taking care of oral health of children (dentists and oral hygienists) should therefore be natural members of interdisciplinary medical support teams around these children. All children, once diagnosed with CHDs, should be referred by the cardiologist for regular oral health visits in order to offer them an individual plan for future dental prevention. Direct comparisons of the dmft/DMFT results in this study with findings from other studies in the same field from other countries should be interpreted with caution because of the different living conditions, varied study settings and designs, and the different methods of control selection used. The current documented high caries prevalence was in accordance with the results from some other caries prevalence epidemiological studies published during the last 10–15 years focusing on this group [10, 14]. However, compared to the present findings, both higher and lower caries prevalence figures in children with CHDs have also been published [15, 16, 35, 36]. Similar to our significantly higher caries experience among cases compared to controls, several studies have also revealed significantly higher caries experience values for children with CHDs than for healthy children [10, 13, 16, 17, 35, 36]. In contrast, some other studies reported similar levels of caries experience in both groups without differences [18–20].

Again, given the use of different indices, caution is also required when comparing results for plaque and gingivitis among different reports. In the present study, gingivitis and plaque were found to be highly prevalent among children with CHDs, and the percentages were much higher than in some of the studies previously mentioned [14, 35, 37]. Fifteen of the cases in the present study were found to have gingival overgrowth. This may be attributed to the use of CHD medications (anti-hypertensive drugs to reduce the pulmonary hypertension caused by some of the CHDs) [38].

Regarding methodology, the response rates for both cases and controls in this study were considered to be excellent [39], indicating that the results can be generalized to the children of the Khartoum state. It was also shown that the mean dmft/DMFT values of the controls in age group 1 and age group 2 were comparable to those in recently conducted surveys among Sudanese children of 3–5 years and 12 years [23, 40, 41], which additionally supports the representativeness of the control group. For the cases, some sub-groups of children with CHDs may have been under-represented, including those who were unable to be referred to the center, those who died because of their condition before being referred to the center, and those who received health care abroad. However, Ahmed Gasim hospital is the referral center that receives the majority of cases for echocardiographic confirmation of their CHD type, even among those who are later sent abroad. To optimize comparability between the case and control groups in age group 2, only the erupted permanent central and lateral incisors and first permanent molars were included, because of the wide range of eruption times of the permanent teeth in the mixed dentition period [42]. Additional strength in the comparisons between groups was given by the higher number of controls than cases. Some of the previously mentioned studies preferred to report the prevalence of caries and gingivitis among children with CHDs without having a control group. They tended to compare their reported dmft/DMFT and GI figures with the figures of the periodic national surveys among school children. Given that recent dmft/DMFT and GI index figures were not available for Sudanese children, controls had to be added to the study for comparison.

It has to be noted that some kappa values were not optimal, and there are therefore reasons to believe that some misclassification may have occurred in the examination process [43]. The WHO criteria for the scoring of caries (as all other visual scoring systems) do not require x-rays and no consideration of bitewings; this expected to have resulted in an underscoring of caries. Furthermore, non-cavitated caries lesions were not scored which means that if we compare it with the International Caries Detection and Assessment System (ICDAS), only the scores from 3 to 6 were recorded. However, from another point of view, WHO caries scoring system, unlike the ICDAS, is more applicable especially in these fieldwork settings where x-rays machines were not available and examinations were to be done in the field without the availability of dental clinics. One more advantage is that this system is so widely used that to some extent enables us for comparisons of data collected by different researchers in different settings and countries.

## Conclusions

Children with CHDs have poor oral health, with high percentages of untreated carious lesions, gingival inflammation, and plaque accumulation in both the primary and permanent dentition. These findings are causes for concern in children at high risk of developing infective endocarditis: their general health is already seriously compromised and it is important to avoid the potential complications of poor oral health. This study provides baseline information necessary for the prioritization of special oral healthcare and free intensive preventive programs within the health system for children with CHDs in Khartoum, Sudan.
